# Biofloc-Based Enhanced Survival of *Litopenaeus vannamei* Upon AHPND-Causing *Vibrio parahaemolyticus* Challenge Is Partially Mediated by Reduced Expression of Its Virulence Genes

**DOI:** 10.3389/fmicb.2020.01270

**Published:** 2020-06-24

**Authors:** Vikash Kumar, Mathieu Wille, Tânia Margarida Lourenço, Peter Bossier

**Affiliations:** ^1^Lab of Aquaculture & Artemia Reference Center, Department of Animal Sciences and Aquatic Ecology, Faculty of Bioscience Engineering, Ghent University, Ghent, Belgium; ^2^ICAR-Central Inland Fisheries Research Institute (CIFRI), Barrackpore, India

**Keywords:** biofloc system, *Litopenaeus vannamei*, *Vibrio parahaemolyticus*, AHPND, phenotype switching

## Abstract

The biofloc system is a relatively new aquaculture technology that offers practical solution to maintain culture water quality by recycling nutrients and improves the health status and resistance of shrimps against microbial infection, yet the mode of action involved remains unclear. This study aimed to unravel the underlying mechanism behind the protective effect of a biofloc system using *Litopenaeus vannamei* and acute hepatopancreatic necrosis disease (AHPND)-causing *Vibrio parahaemolyticus* M0904 strain as a host-pathogen model. The results showed that a biofloc system maintained at a C/N ratio of 15, improves the water quality and contributes to the nutrition of cultured animals as bioflocs might serve as an additional protein source. Furthermore, the study demonstrated that the biofloc system enhances the survival of *L. vannamei* upon challenge with a *V. parahaemolyticus* AHPND strain. Remarkably, the results highlight that in the biofloc system, AHPND-causing *V. parahaemolyticus* possibly switch from free-living virulent planktonic phenotype to a non-virulent biofilm phenotype, as demonstrated by a decreased transcription of flagella-related motility genes (*flaA*, *CheR*, and *fliS*), Pir toxin (*PirB*^*VP*^), and AHPND plasmid genes (*ORF14*) and increased expression of the phenotype switching marker *AlkPhoX* gene in both *in vitro* and *in vivo* conditions. Taken together, results suggest that biofloc steer phenotype switching, contributing to the decreased virulence of *V. parahaemolyticus* AHPND strain toward shrimp postlarvae. This information reinforces our understanding about AHPND in a biofloc setting and opens the possibility to combat AHPND not only by trying to eliminate the AHPND-causing *V. parahaemolyticus* from the system but rather to steer the phenotypic switch.

## Introduction

Shrimp aquaculture is one of the fastest growing high-protein animal food producing sectors in the world (FAO, 2014; Kumar et al., 2016; Roy et al., 2018). However, disease outbreaks, causing production loss in shrimp aquaculture, have moved to the forefront in recent years. Particularly in Asia and North America, acute hepatopancreatic necrosis disease (AHPND) has resulted in collective losses exceeding an estimated US $43 billion (China, Malaysia, Thailand, Vietnam, and Mexico; Leung and Bates, 2013; Shinn et al., 2018; Kumar V. et al., 2018; Roy et al., 2019). Multiple factors might be at the basis of AHPND. The presence of virulent *V. parahaemolyticus* (VP_*AHPND*_) is the primary factor (Kumar and Bossier, 2018, 2019; Roy et al., 2020). However, a compromised health status of the cultured animals in combination with suboptimal environmental conditions most likely facilitates AHPND outbreaks, resulting in high mortalities of juvenile shrimp and often entire loss of stocks within 30 days of stocking (Liu and Chen, 2004; Sajali et al., 2019; Kumar et al., 2019b). Therefore, disease prevention and control measures should not only focus on maintaining a biosecure environment but should be based on an integral approach ensuring among others adequate nutrition and good health of the aquatic animals and maintenance of optimum water quality.

Growing shrimp in a biofloc system can be a promising alternative strategy to improve environmental conditions and health status of cultured animals. The basic principle of the biofloc system is to recycle waste nutrients, in particular, inorganic nitrogen resulting from uneaten feed and feces into microbial biomass, by steering the C/N ratio of the water through the modification of carbohydrate content in feed or by addition of a carbon source in the water (Avnimelech, 1999; Crab et al., 2012; Ekasari et al., 2014; Kumar V.S. et al., 2018). Biofloc systems have been reported to increase feed utilization, growth, and survival. Biofloc can also act as immunostimulants to enhance the shrimp innate immune system and provide protection against microbial pathogens including AHPND-causing *V. parahaemolyticus* (Crab et al., 2010a; Kim et al., 2014; Bossier and Ekasari, 2017; Sajali et al., 2019). In fact, bioflocs as such can decrease the impact of AHPND *V. parahaemolyticus* challenge; however, the protective potential depends on operational parameters of the biofloc system (Hostins et al., 2019). In this context, there is an urgent need to systematically unravel the protective mechanisms of a biofloc environment and develop a biofloc system that can control AHPND infection in shrimp aquaculture.

In the previous studies, it was found that the M0904 AHPND strain contains a pVA1 plasmid that harbor the PirAB^*VP*^ toxin genes and encodes for the binary toxins named PirA^*VP*^ and PirB^*VP*^. The binary PirAB^*VP*^ toxin are the primary virulence factor of *V. parahaemolyticus* that mediate AHPND etiology and mortality in shrimps (Kumar V. et al., 2018; Kumar et al., 2019a, b; Roy et al., 2019). However, in the subsequent study it was shown that environmental conditions can change the *V. parahaemolyticus* M0904 strain phenotype, displaying, among others, strongly down-regulated expression of flagella-related motility flagellin (*flaA*), chemotaxis protein (*CheR*), flagellin specific chaperone (*fliS)*, and virulence related genes (Pir toxin, *PirB*^*VP*^, and AHPND plasmid coding gene, *ORF-14*), while the expression of phenotype switching marker, alkaline phosphatase PhoX (*AlkPhoX*) gene, was significantly increased (Kumar et al., 2020). In addition, the phenotype switching resulted in decreased virulence of AHPND-causing *V. parahaemolyticus* strain toward two crustacean larvae, namely *Artemia* and *Macrobrachium*.

Based on observations on the one hand of *V. parahaemolyticus* phenotype switching in altered environmental conditions (Kumar et al., 2020) and results of Hostins et al. (2019) on the other hand on protective potential of biofloc system against AHPND-causing *V. parahaemolyticus*, it is hypothesized that a biofloc environment is involved in setting the phenotypic status of AHPND-causing *V. parahaemolyticus* under both *in vitro* and *in vivo* conditions, altering its virulence. This hypothesis was verified using a biofloc-based *L. vannamei* and *V. parahaemolyticus* model. The findings demonstrate that apart from improving water quality and growth performance of shrimp, the described biofloc system indeed steers phenotype switching in the *V. parahaemolyticus* AHPND strain and this effect leads to a decreased *in vitro* and *in vivo* virulence and an improved survival of *L. vannamei*.

## Materials and Methods

### Experimental Design

The experiments were carried out at the Laboratory of Aquaculture & *Artemia* Reference Center, Ghent University, Belgium. Six glass aquaria tanks (39 cm × 21 cm × 25 cm) filled with 20 L seawater (30 g l^–1^ salinity) were used as the experimental culture units. During the entire culture period, the photoperiod was maintained with a regime of 12 h light and 12 h darkness, and the water temperature was maintained at 27.5–28.5°C in a controlled temperature room. The Pacific white shrimp postlarvae (*Litopenaeus vannamei*) obtained from Shrimp Improvement Systems (SIS, Miami, United States), previously conditioned and acclimatized to the experimental condition for 1 week, at an initial average body weight of 0.31 ± 0.02 *g*, were randomly distributed in the tanks at a density of 30 shrimps/tank (366 shrimps m^–2^). Four times daily, a commercial pellet diet containing 35% crude protein and 10% crude fat (CreveTec Grower 2, Belgium) was provided for 28 days to all the tanks. The feeding levels were determined at 7% on wet body weight per day and the daily feed amount was adjusted to the biomass in the tanks.

For biofloc development, the biofloc starter culture, e.g., water sample (100 ml) from an established biofilter system, were collected and added to 100 l seawater (30 g l^–1^ salinity). Subsequently, the carbon source [D(+)-glucose, Merckmillipore, Belgium] was added continuously throughout the day to provide an estimated daily input C/N ratio of 15 [based on the previous work from our laboratory by Hostins et al. (2019), demonstrated to provide protection against AHPND at C/N ratio of 15], to induce the biofloc formation (Crab et al., 2010a, b; Hostins et al., 2019). The C/N ratio was calculated based on the following assumptions: the commercial feed contains 35–40% protein; 16% of protein is nitrogen (*N*); approximately 75% of ingested *N* is excreted by the shrimp and 50% of glucose is carbon.

The amount of glucose (*g*) added to maintain C/N ratio of 15 were calculated with the following formula

Glucose (*g*): Feed input (*g*) × 0.35 (protein content) × 0.16 (*N* in feed) × 0.75 (*N* excreted) × 15 × 2

After 2 days, the biofloc water (BFW) was distributed in treatment tanks and the control tanks were filled with seawater (30 g l^–1^ salinity), in three replicates (Supplementary Figure S1; De Schryver et al., 2008; Hostins et al., 2019). Afterwards, during 28-days culture period, glucose (carbon source) was added daily 2 h after feeding at an estimated C/N ratio of 15 to maintain homogeneous biofloc in the treatment group. The culture water in the control group was exchanged daily @ 60% with seawater (30 g l^–1^ salinity); in the treatment group seawater was regularly added only to make up for the water loss due to evaporation.

### Water Quality

The dissolved oxygen, temperature, pH, and salinity were daily measured *in situ* using a portable multiparameter photometer (Hanna instruments, Belgium), pH meter (VWR, Belgium), and refractometer (VWR, Belgium). The dissolved inorganic nitrogen, i.e., nitrite nitrogen (NO_2_^–^-N) and nitrate nitrogen (NO_3_^–^-N), were determined every 2 days using a multiparameter photometer (Hanna instruments, Belgium) according to the manufacturer instructions. On every second day, the BFW samples were filtered using 0.6-μm glass fiber micro-filters (GF-6, Macherey-Nagel, Düren, Germany) and were monitored for total suspended solids (TSS; APHA, 2005). The weight difference of the dried samples before and after ignition was taken for calculation of volatile suspended solids (VSS). The biofloc volume was also measured every second day after 15–20 min of sedimentation using Imhoff cones. The Kjeldahl nitrogen (Kj-N) and total ammonia nitrogen (TAN; NH_4_^+^-N + NH_3_) were measured according to standard methods (Crab et al., 2007). The difference between Kj-N and TAN was used to calculate the protein content of the biofloc by multiplying the organic nitrogen content by 6.25 (Jauncey, 1982; Crab et al., 2010a).

### Growth Performance

The growth performance of *L. vannamei* cultured in either biofloc system or clear water system was analyzed at the end of the experimental period. The wet weight of shrimps from the treatment and control groups were measured and performance was assessed using the following formula:

Weight gain (%) = (Final weight – Initial weight)/Initial weight × 100
Weekly weight gain (g wk^–1^) = (Final weight – Initial weight)/week
SGR (specific growth rate; %) = (ln average final weight – ln average initial weight)/number of culture days × 100
FCR (feed conversion ratio) = Total dry feed intake (g)/wet weight gain

### Challenge Assay

**FIGURE 1:**
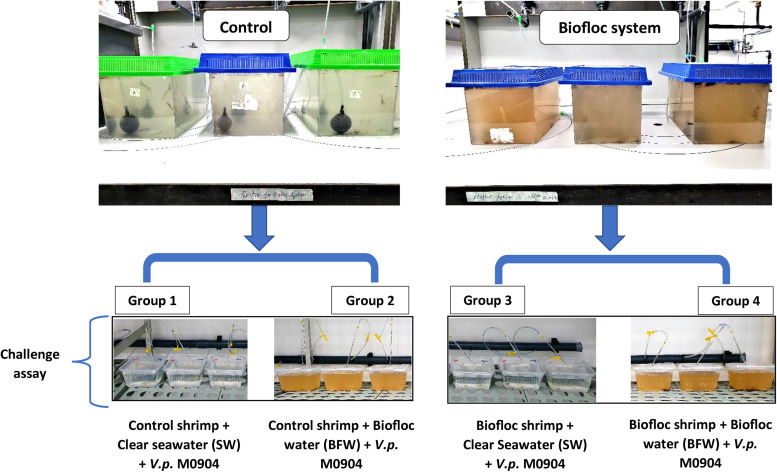
Schematic of *Litopenaeus vannamei* postlarvae challenge assay grown in control clear water and biofloc water and challenged with *V. parahaemolyticus* M0904 strain.

To determine the protective effect of bioflocs in *L. vannamei* postlarvae, a challenge assay was performed in four separate groups (Figure 1). Briefly, the shrimp postlarvae cultured in either the biofloc system or the clear water system for 28 days were collected and transferred to 2 L plastic containers. In the first and second group, 30 shrimp postlarvae from clear water control group were collected and group of 5 postlarvae/replicate were transferred to 2 L plastic container filled with either 1 L clear seawater (SW; 30 g l^–1^ salinity) or 1 L BFW. Similarly, in the third and fourth group, the 30 shrimp postlarvae collected from biofloc system were transferred to 2 L plastic container and filled with either 1 L SW or BFW. All the containers provided with aeration was maintained at 27.5–28.5°C in a controlled temperature room. Subsequently, it was verified whether the addition of BFW could protect the postlarvae against subsequent challenge, by immersion, with *V. parahaemolyticus* M0904 strain (BCCM accession number – LMG P-31518) at 10^6^ cells/ml. The day before challenge assay, 50 μl of *V. parahaemolyticus* M0904 strain stock culture was inoculated into 20 ml sterile marine broth in 50 ml erlenmeyer and grown overnight at 28°C under constant agitation (120 rpm). The survival of shrimp postlarvae was scored at 12, 24, 36, and 48 h after the addition of the pathogen. The postlarvae that were not challenged with *V. parahaemolyticus* were maintained as negative controls. Each group in the treatment and control were maintained in triplicate.

### RNA Extraction From Biofloc and Clear Seawater (*in vitro*) Supplemented With *Vibrio parahaemolyticus*

The water from either biofloc system or the clear water system, collected in separate 2 L plastic containers in triplicates, were supplemented with the *V. parahaemolyticus* M0904 strain @ 10^6^ cells/ml and further incubated at 28°C. After 12 and 24 h of incubation, the microbial cells were harvested through centrifugation (4000×*g* for 15 min), and total RNA was extracted with Qiagen RNeasy Plus Mini Kit (Cat No. 74136) according to the manufacturer instructions. The RNA quality and quantity were measured on NanoDrop spectrophotometer (ThermoFisher Scientific, Belgium) and RNA samples with A260/A280 ratios > 2.0 and A260/A230 ratios > 1.5 were used for the analysis. The RNA integrity was checked by agarose gel electrophoresis and the RNA samples were stored in −80°C for subsequent use.

### Bacterial RNA Extraction From Challenged Shrimp Postlarvae

The bacterial RNA was extracted from challenged *L. vannamei* postlarvae as described previously (Ruwandeepika et al., 2015). The Pacific white shrimp cultured for 28 days in bioflocs water were challenged with AHPND causing *V. parahaemolyticus* M0904 strain (@ 10^6^ cells/ml) in new seawater (BFW to SW) or in their respective bioflocs water (BFW to BFW). Similarly, the shrimp cultured in seawater were challenged with AHPND causing *V. parahaemolyticus* in new seawater (SW to SW) or in bioflocs water (SW to BFW). After 12 and 24 h of incubation, the shrimp were washed with sterile seawater and the hepatopancreas of 3 individual shrimp/group were separately dissected, following a standard protocol of hepatopancreas collection (Cervellione et al., 2017a, b), frozen immediately in liquid nitrogen, and after that they were stored at -80°C until RNA extraction. Before RNA extraction, the postlarval hepatopancreas were homogenized using eppendorf grinder (Mini-Beadbeater, BioSpec Products, United States) under aseptic conditions. Afterwards, the suspension was centrifuged (4000 × *g* for 5 min) and supernatant was collected to avoid the clogging of the RNA extraction columns during the subsequent RNA extraction. Afterward, the extraction of total RNA was performed as described above for the RNA extraction from *in vitro* BFW.

### Reverse Transcription

Reverse transcription was done with the RevertAid^TM^ H Minus First Strand Complementary deoxyribonucleic acid (cDNA) Synthesis Kit (Thermo Fisher Scientific, Belgium) according to the manufacturer’s guidelines. Briefly, 1 μg total RNA, and 1 μl random hexamer primer solution was mixed first. Then, 8 μl of reaction mixture containing 4 μl of 5× reaction buffer (250 mM Tris–HCl pH 8.3, 250 mM KCl, 20 mM MgCl_2_, and 50 mM DTT), 2 μl of 0.01 mol^–1^ dNTP mix, 20 units of ribonuclease inhibitor, and 200 units of RevertAid^TM^ H minus M-MuLV reverse transcriptase was added. The reaction mixture was incubated for 5 min at 25°C followed by 60 min at 42°C. The reaction was terminated by heating at 70°C for 5 min and then cooled to 4°C. cDNA samples were checked by PCR and stored at −20°C for further use.

### Quantitative Real-Time PCR (RT-QPCR) Analysis

The expressions of 3 flagella-related motility genes, including regulators, structural, and chemotaxis genes, 2 VP_*AHPND*_ plasmid related virulent genes including toxin and coding genes of virulent plasmid and *AlkPhoX* gene, marker gene that indicates the presence of non-virulent AHPND *V. parahaemolyticus* phenotype, were measured by RT-qPCR with pair of specific primers using StepOnePlus Real-time PCR systems (Applied Biosystems; Supplementary Table S1; Han et al., 2015; Yang and Defoirdt, 2015; Kumar et al., 2020). The Ct values from the two reference genes *rpoA* (RNA polymerase A submit) and *toxR* mRNA, used as the internal control, were subjected to geomean and expression of the genes was calculated relative to the *rpoA* and *toxR* mRNA levels. The amplification was performed in a total volume of 20 μl, containing 10 μl of 2× Maxima SYBR Green/ROX qPCR Master Mix (Thermo Fisher Scientific), 1 μl of cDNA (50 ng), 8 μl of nuclease free water, and 0.5 μl of each specific primer. Master mixes were prepared for each biological replicate of the sample in triplicate and RT-qPCR for target and reference genes was performed with a four-step amplification protocol: initial denaturation (10 min at 95°C); 40 cycles of amplification and quantification (15 s at 95°C, 30 s at 60°C, and 30 s at 72°C); melting curve (55–95°C) with a heating rate of 0.10°C/s and a continuous fluorescence measurement, and cooling (4°C). A negative control reaction was included for each primer set by omitting template cDNA. The comparative CT method (2-ΔΔCt method) following Livak and Schmittgen (2001) was used to analyze the expression level of the target genes and verified by Pfaffl relative standard curve method (Pfaffl, 2002). The Log transformed 2^ΔΔCT value were subjected to a *t*-test, and the *P* values smaller than 0.05 were considered statistically significant.

### Statistical Analysis

Water quality and growth parameter data were arcsin transformed to satisfy normality and homoscedasticity requirements as necessary. The data were then subjected to one-way analysis of variances (ANOVA) followed by Duncan’s multiple range test using statistical software statistical package for the social sciences version 24.0. *P* values ≤ 0.01 were considered significant. Survival data of *L. vannamei* were subjected to logistic regression analysis using GenStat 16 (VSN International, Hemel Hempstead, United Kingdom) to determine significant differences between the control and treatment. Gene expressions results were represented as fold expression relative to the geometrical mean of two internal control genes (*toxR* and *rpoA*). The expression level in the control clear SW group (+*V. parahaemolyticus*) was regarded as 1.0 and thereby the expression ratio of the treatment biofloc (BFW) group (+*V. parahaemolyticus*) was expressed in relation to the SW group in the *in vitro* biofloc experiment. Moreover, in the *in vivo* experiment, the expression level in the SW to SW group was regarded as 1.0 and thereby the expression of the SW to BFW, BFW to SW, and BFW to BFW was expressed in relation to the SW to SW group. Statistical analysis for the significant differences in the expression levels between the control and treatment groups was performed with single-tailed Student’s *t*-tests using the log transformed data.

## Results

### Biofloc Improves Water Quality and Growth Performance of *L. vannamei*

In the first experiment, the water quality of biofloc system was monitored every week for a period of 28 days and was compared with that in the clear water system (Table 1). Significantly lower values of DO and pH were recorded in BFW as compared to the clear water system. The dissolved inorganic nitrogen concentration (NH_4_^+^-N and NO_2_^–^-N) was significantly lower in BFW as compared to clear water system (Table 1). However, higher values of NO_3_-N were observed in BFW as compared with clear water system in all sampling weeks. The TSS and VSS values were recorded significantly higher in biofloc group as compared to clear water control group. A crude protein content between 28–43% was observed in biofloc group during the sampling weeks (Table 1). These results indicate that in a biofloc system maintained at a calculated C/N ratio of 15, water quality is improved.

**TABLE 1:** Mean values of water quality parameters in control and biofloc groups (mean ± SE).

**Water quality parameters**		**7 days**	**14 days**	**21 days**	**28 days**
TAN (NH_4_^+^-N + NH_3_; mg l^–1^)	Control	0.420.14^b^	0.820.11^b^	10.20^b^	0.640.12^b^
	Treatment	0.170.025^a^	0.450.14^a^	0.500.20^a^	0.430.12^a^
Nitrite (NO_2_^–^-N; mg l^–1^)	Control	0.60.23^b^	1.370.23^b^	0.820.16^a^	0.570.11^a^
	Treatment	0.420.21^a^	0.30.11^a^	0.570.16^a^	0.520.12^a^
Nitrate (NO_3_^–^-N; mg l^–1^)	Control	1.620.55^a^	2.120.42^a^	2.350.33^a^	2.220.13^a^
	Treatment	121.22^b^	502.04^b^	61.52.21^b^	64.752.05^b^
Total suspended solids (TSS; mg l^–1^)	Control	62.972.34^a^	51.852.36^a^	64.321.56^a^	70.721.97^a^
	Treatment	171.723.59^b^	161.824.02^b^	164.222.89^b^	176.63.64^b^
Volatile suspended solids (VSS; mg l^–1^)	Control	13.270.61^a^	10.820.43^a^	12.50.27^a^	12.920.38^a^
	Treatment	54.023.25^b^	56.572.64^b^	59.251.98^b^	58.673.68^b^
pH	Control	8.170.08^b^	8.370.062^b^	8.280.048^b^	8.420.085^b^
	Treatment	7.350.06^a^	7.250.028^a^	7.40.081^a^	7.320.11^a^
Dissolved Oxygen (mg l^–1^)	Control	6.650.11^b^	6.50.14^b^	6.70.05^b^	6.70.09^b^
	Treatment	5.50.12^a^	5.470.12^a^	5.60.15^a^	5.420.085^a^
Temperature (°C)	Control	28.30.13^a^	28.50.12^a^	28.20.11^a^	28.30.14^a^
	Treatment	28.50.12^a^	28.40.14^a^	28.30.14^a^	28.50.11^a^
Salinity (g l^–1^)	Control	30	30	30	30
	Treatment	30	30	30	30
Crude protein (% dry weight)	Control	0	0	0	0
	Treatment	28.720.28	42.571.52	44.972.99	43.681.85

*Significant differences between control and treatment (biofloc) groups at each sampling point are indicated with different superscript (*n* = 9). Different superscript letters for a specific parameter indicate significant differences.*

Next, to determine whether the rearing of *L. vannamei* in biofloc system improves the nutrition and growth of the cultured animals, the growth parameters of shrimp after 28 days of culture period were analyzed. The results showed that growth performance of shrimp reared in biofloc system was significantly higher as compared to those of shrimp cultured in clear water system (Table 2). Final body weight, weight gain, weekly weight gain, and specific growth rate of shrimp reared in the biofloc system were significantly increased as compared to those cultured in clear water system. Additionally, the biofloc maintained at C/N ratio of 15 resulted in significantly lower food conversion ratio (FCR) as compared to the control (Table 2). These results suggest that biofloc is an effective method to improve culture environment and nutrition of the cultured shrimp and the beneficial effect of biofloc is paralleled with improved water quality and growth performance of shrimp in biofloc system (Ekasari et al., 2014; Luis-villaseñor et al., 2016; Bossier and Ekasari, 2017; Lee et al., 2017).

**TABLE 2:** Mean values of growth parameters in control and biofloc groups (mean ± SE).

**Parameters**	**Control**	**Treatment**	***p* < 0.01**
Initial weight (g)	0.310.022^a^	0.310.022^a^	Non-significant
Final weight (g)	1.730.044^a^	2.180.05^b^	Significant
Weight gain (g)	1.430.055^a^	1.860.042^b^	Significant
Weekly weight gain (g wk^–1^)	0.360.013^a^	0.470.01^b^	Significant
Specific growth rate (% day^–1^)	4.790.18^a^	6.210.14^b^	Significant
FCR	1.490.01^b^	1.210.06^a^	Significant

*Significant differences between control and treatment (biofloc) groups are indicated with different superscript. Different superscript letters for a specific parameter indicate significant difference.*

### Biofloc Conferred Protection to *L. vannamei* Postlarvae Against Subsequent *V. parahaemolyticus* AHPND Strain Challenge

**FIGURE 2:**
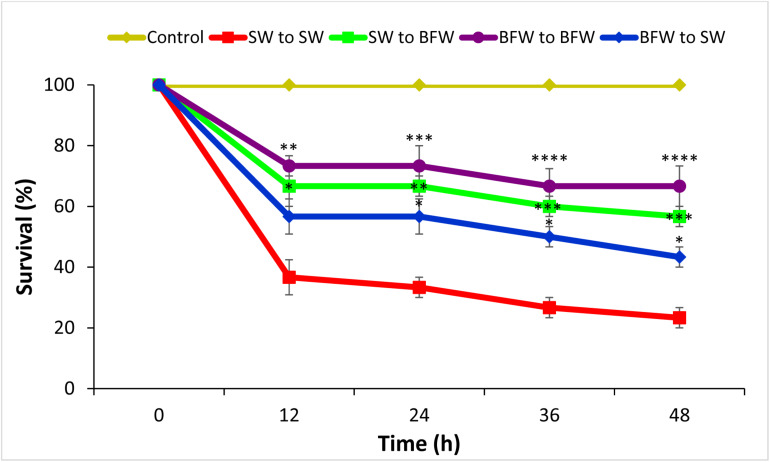
Effect of Biofloc on the survival of *Litopenaeus vannamei* postlarvae after 12, 24, 36, and 48 h post challenge with *V. parahaemolyticus* M0904 strain. The Pacific white shrimp cultured for 28 days in bioflocs water were challenged with AHPND causing *V. parahaemolyticus* (@ 10^6^ cells/ml) in new seawater (BFW to SW) or in their respective bioflocs water (BFW to BFW). Similarly, the shrimp cultured in seawater were challenged with AHPND causing *V. parahaemolyticus* in new seawater (SW to SW) or in bioflocs water (SW to BFW). The postlarvae that were not challenged with *V. parahaemolyticus* served as negative controls. Error bars represent the standard error of three replicates and asterisks represents significant difference between the SW to SW with SW to BFW, BFW to SW, and BFW to BFW *(*P* < 0.05), **(*P* < 0.01), ***(*P* < 0.001), and ****(*P* < 0.0001).

Next, the potential role of BFW in conveying protection in shrimp against AHPND was evaluated. It was verified by four different approaches as demonstrated in Figure 2. The results showed that *L. vannamei* postlarvae reared for 28 days in biofloc system display significantly higher survival (group 2, 3, and 4) compared to the clear seawater shrimp postlarvae (group 1). In fact, the protective effect against *V. parahaemolyticus* for biofloc reared shrimp even lasted when transferred to clear water (group 3; Figure 2). Additionally, the results also showed that *L. vannamei* reared in clear seawater system but challenged in the presence of flocs are also protected (Figure 2), indicating that the protective effect can be conveyed by bioflocs as such.

### Biofloc Steer Phenotype Switching in *V. parahaemolyticus* AHPND Strain and Modulate *in vitro* Expression of Virulence Related Genes

**FIGURE 3:**
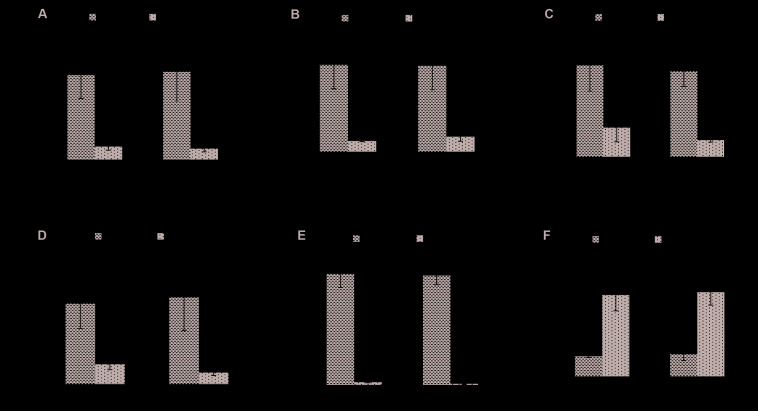
Expression of flagella-related motility genes: **(A)** *flaA* (polar flagellin), **(B)** *flis* (polar flagellin specific chaperone), **(C)** *cheR* (chemotaxis protein); virulent AHPND plasmid genes: **(D)** *ORF14*, **(E)** *PirB*^*VP*^, and a marker gene that indicate presence of non-virulent AHPND *V. parahaemolyticus* phenotype: **(F)** *AlkPhoX* (alkaline phosphatase PhoX) from *in vitro* experiment. The expression level in the control clear water group (+ *V. parahaemolyticus*) was regarded as 1.0 and thereby the expression ratio of the biofloc group (+ *V. parahaemolyticus*) was expressed in relation to the control SW group (+ *V. parahaemolyticus*). The results are the mean ± SE (*n* = 3) and are presented relative to *V. parahaemolyticus toxR* and *rpoA* mRNA. Asterisks represents significant difference between the control SW group and biofloc group (+ *V. parahaemolyticus*) ^∗^(*P* < 0.05), ^∗∗^(*P* < 0.01), ^∗∗∗^(*P* < 0.001), ^****^(*P* < 0.0001), and ^*****^(*P* < 0.00001).

To investigate the effect of biofloc system further, *in vitro* temporal expression of virulence-related genes, i.e., flagella-related motility genes, including polar *flaA*, *CheR*, polar *fliS* gene, and VP_*AHPND*_ plasmid related virulent genes, including toxin (*PirB*^*VP*^) and coding genes of the virulent plasmid (*ORF14*), were investigated from control clear SW (+*V. parahaemolyticus*) and treatment BFW (+*V. parahaemolyticus*). As for the flagella-related motility genes, there was a significant decrease in the expression of *flaA*, *CheR*, and *flis* genes in the BFW as compared to the SW group at any of the time point tested (Figures 3A–C). In addition, the transcript level of *PirB*^*VP*^ and *ORF14* was also markedly decreased in the BFW when compared with the SW group at 12 and 24 h post treatment (Figures 3D,E).

Next, the differential expression of the phenotype switching marker *AlkPhoX* gene was determined from SW and BFW. Interestingly, as shown in Figure 3F, the mRNA transcript levels of *AlkPhoX* gene in the BFW increased significantly as compared to SW group (+*V. parahaemolyticus*) at 12 and 24 h post treatment. This observation suggests that the biofloc environment indeed facilitates phenotypic switching in *V. parahaemolyticus* leading to a decreased transcription of virulence related genes and increased expression of phenotype switching marker gene, *AlkPhoX* gene (Kumar et al., 2020).

### Phenotype Switching of *V. parahaemolyticus* in Biofloc System Attenuates the *in vivo* Expression of Virulence Related Genes in *L. vannamei*

**FIGURE 4:**
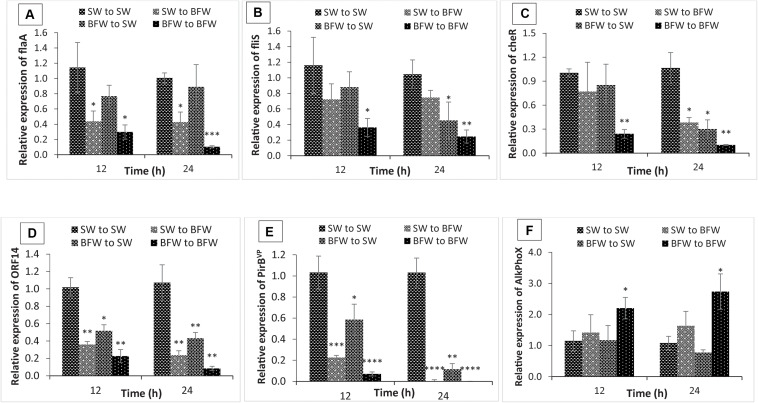
Expression of flagella-related motility genes: **(A)** *flaA* (polar flagellin), **(B)** *flis* (polar flagellin specific chaperone), **(C)** *cheR* (chemotaxis protein); virulent AHPND plasmid genes: **(D)** *ORF14*, **(E)** *PirB*^*VP*^, and marker gene that indicate presence of non-virulent AHPND *V. parahaemolyticus* phenotype: **(F)** *AlkPhoX* (alkaline phosphatase PhoX) from shrimp *in vivo* experiment. The gene expression level in Pacific white shrimp cultured in seawater and subsequently challenged with AHPND-causing *V. parahaemolyticus* in new seawater (SW to SW) was regarded as 1.0. Thereby the expression of SW to BFW, BFW to SW, and BFW to BFW was expressed in relation to the SW to SW. The results are the mean ± SE (*n* = 3) and are presented relative to *V. parahaemolyticus toxR* and *rpoA* mRNA. Asterisks represents significant difference between the SW to SW with SW to BFW, BFW to SW, and BFW to BFW *(*P* < 0.05), **(*P* < 0.01), ***(*P* < 0.001), ****(*P* < 0.0001), and *****(*P* < 0.00001).

To study further the transcriptional modifications occurring in biofloc system, the *in vivo* temporal expression of flagella-related motility genes, VP_*AHPND*_ plasmid related virulent genes and phenotype switching marker gene of *V. parahaemolyticus* AHPND strain from control shrimp (SW group) in clear seawater (SW to SW; +*V. parahaemolyticus*) were investigated and compared with control shrimp in biofloc water (SW to BFW; +*V. parahaemolyticus*) and biofloc shrimp in either BFW to SW or BFW to BFW (+*V. parahaemolyticus*). Similar to the *in vitro* experiment, the transcription of flagella-related motility genes, i.e., flaA, CheR, and flis genes and VP_*AHPND*_ plasmid related virulent genes, *PirB*^*VP*^ and *ORF14*, was significantly decreased in *L. vannamei* at 12 and 24 h post exposure (+*V. parahaemolyticus*) in BFW to SW or BFW to BFW (+*V. parahaemolyticus*) as compared to the SW to SW group (Figures 4A–E). In addition (Figure 4), there was a downregulation of *flaA*, *CheR*, *flis*, *PirB*^*VP*^, and *ORF14* gene in SW to BFW group (+*V. parahaemolyticus*) when compared with the SW to SW group (+*V. parahaemolyticus*).

To further substantiate that biofloc system induces phenotypic switching and increase the expression of the switching marker gene, the transcription of *AlkPhoX* gene was examined in shrimp reared under biofloc or clear water conditions. Interestingly, increased expression level of *AlkPhoX* gene was observed at 12 and 24 h post exposure (+*V. parahaemolyticus*) in BFW to BFW group as compared to the SW to BFW, BFW to SW, and SW to SW groups (Figure 4F). In contrast, downregulation of *AlkPhoX* gene was recorded in BFW to SW group after 24 h post challenge (+*V. parahaemolyticus*). Taken together, our *in vivo* assay strongly suggests that the biofloc system indeed plays an essential role in phenotype switching, mediating virulence of *V. parahaemolyticus* in *L. vannamei* postlarvae.

## Discussion

Acute hepatopancreatic necrosis disease, a newly emergent farmed penaeid shrimp bacterial disease originally known as early mortality syndrome (EMS), has been particularly devastating in the cultivation of shrimp, causing massive mortality (up to 100%) within 20–30 days of post-larvae stocking (Tran et al., 2013; Lee et al., 2015; Dong et al., 2017; Kumar V. et al., 2018; Kumar et al., 2019a, b; Roy et al., 2019). However, the AHPND *V. parahaemolyticus* strain, under differential flow conditions (low fluid shear stress), switches phenotype causing a major shift in the protein secretome, e.g., *AlkPhoX* is produced instead of PirA^*VP*^/PirB^*VP*^ toxins. This phenotype is also less virulent to shrimp species (Kumar et al., 2020). On the other hand it has been shown that bioflocs stimulates the innate immunity and decrease the impact of AHPND *V. parahaemolyticus* challenge in shrimp (Hostins et al., 2019). In combination, these observations suggest that the protective mechanisms of biofloc might be at least, in part, related to the specific biofloc environment that potentially regulates the virulence of AHPND *V. parahaemolyticus*. Here, a possible scenario is considered that biofloc environment is involved in mediating the phenotypic status of AHPND-causing *V. parahaemolyticus*. In addition, it is assumed that biofloc induced phenotype switching modulates the *in vitro* and *in vivo* virulence of AHPND strain.

Biofloc technology offers interesting perspectives of high-density culture with the possibility to maintain good water quality with minimal or no water exchange by nutrient recycling, in particular, nitrogen into microbial biomass that can be used *in situ* by the cultured animals (Avnimelech, 1999; Kuhn et al., 2009; Fatimah et al., 2019). In the present study, the water quality characteristics in the biofloc group were significantly improved and found to be within the optimal range required for growth and improved immunity of *L. vannamei* (Wickins, 1976; Cohen et al., 2005; Mishra et al., 2008). TAN (NH_4_^+^-N + NH_3_) and nitrite (NO_2_–N) concentration were significantly reduced in the biofloc system after 7, 14, 21, and 28 days culture period as compared to the control (Table 1). In addition, the biofloc system significantly improved the growth performance and *L. vannamei* tolerance against the AHPND-causing *V. parahaemolyticus* strain (Table 2 and Figure 2). The biofloc system is generally formed by aggregation of microorganism, microalgae, organic particles, or solids from uneaten feeds (Cohen et al., 2005; Wasielesky et al., 2006; Azim and Little, 2008; De Schryver et al., 2008; Ju et al., 2008; Trichet, 2010; Cardona et al., 2015; Lee et al., 2017). Also, for shrimp in a biofloc system, flocs consumed may constitute up to 29% of the daily feed intake (Burford et al., 2003). Therefore, the consumed biofloc might nutritionally modulate the shrimp health status resulting in improved growth performance and increased protection of *L. vannamei* against *V. parahaemolyticus* AHPND strain.

Shear stress or flow behavior is an important factor modulating the growth of bacteria either in a free-living planktonic form or in a matrix enclosed communities (e.g., a biofilm; Crabbé et al., 2008; Giese et al., 2014; Mancilla et al., 2015; Dingemans et al., 2016; Kumar et al., 2020). Hence, to gain insight into the *V. parahaemolyticus* phenotype status in biofloc system, we used qPCR technique to assess the *in vitro* temporal expression of flagella-related motility genes and VP_*AHPND*_ plasmid related virulent genes. The results indicated that expression of polar *flaA*, *CheR*, and polar *fliS* gene was significantly decreased in biofloc group (+*V. parahaemolyticus*) when compared with the control SW group (+*V. parahaemolyticus*; Figures 3A–C). Since there exist a correlation between flagellar motility and virulence of bacterial pathogens, the temporal downregulation of flagellar motility genes in *V. parahaemolyticus* suggests a decrease in its virulence in biofloc condition (Watnick, 2000; Hawkins et al., 2017; Zhang et al., 2018). In addition, the transcription of pVA1 plasmid-bound genes, i.e., *PirB*^*VP*^ (responsible for PirB^*VP*^ toxin), and *ORF-14* (copy number of the pVA1 plasmid) which mediate the virulence of *V. parahaemolyticus* was also significantly downregulated in the biofloc group (+*V. parahaemolyticus*) as compared with the control SW group (+*V. parahaemolyticus*; Figures 3D,E). In contrast, the *in vitro* temporal expression of *AlkPhoX* gene in the biofloc group (+*V. parahaemolyticus*) was significantly increased at 12 and 24 h as compared to the control SW group (+*V. parahaemolyticus*; Figure 3F). The results therefore suggest that *V. parahaemolyticus* in biofloc system switch to non-virulent phenotype, as deduced from the increased expression of phenotype switching marker gene, *AlkPhoX* gene, with a simultaneous decrease of the expression of flagella-related motility and other virulent genes. This expression profile has previously been shown to be associated with the non-virulent phenotypic status of this *V. parahaemolyticus* AHPND strain (M0904 strain; Kumar et al., 2020).

The hepatopancreas is considered the primary target tissue of AHPND bacteria, as affected shrimp show an abnormal hepatopancreas (small, shrunken, or black coloration) and histopathological lesions on gross examination (Eduardo and Mohan, 2012; Lightner et al., 2013), apparently caused by *in situ* PirA^*VP*^/PirB^*VP*^ toxin production. Hence, *L. vannamei* hepatopancreas was used to study the *in vivo* effect of biofloc supplementation on the phenotype status and virulence of AHPND bacteria by measurement of flagella-related motility genes and VP_*AHPND*_ plasmid related virulent genes expression after 12 and 24 h post AHPND-causing *V. parahaemolyticus* challenge. In the control clear SW group, *V. parahaemolyticus* produce AHPND-causing Pir toxin in the hepatopancreas, and hence appear to adopt the planktonic mode of growth (Figures 4A–E; Nunan et al., 2014; Khimmakthong and Sukkarun, 2017). However, in the treatment biofloc group, the expression of motility and Pir toxin genes in the hepatopancreas were significantly downregulated, and increased production of *AlkPhoX* was recorded (Figure 4F). The results allow to develop the following hypothesis. When AHPND-causing *V. parahaemolyticus* are added into biofloc system, the bacterium might get settled in flocs and possibly switch into the biofilm phenotype (*in vitro* upregulation of *AlkPhoX* gene, found in earlier results). Subsequently, after consumption of flocs, the AHPND bacteria enters the host. Additionally, the production of *AlkPhoX* in hepatopancreas of biofloc treatment shrimps suggests that under biofloc consumption conditions *V. parahaemolyticus* might remain in the biofilm phenotype and is not switching back to the planktonic status. It is, however, not clear why AHPND *Vibrio* inside the host don’t switch back to the free-living planktonic form and produce PirA^*VP*^/PirB^*VP*^ toxins, like it seems to happen when AHPND symptoms are apparent. This needs further investigations. Taking together, it can be suggested that biofloc indeed induces phenotype switching in *V. parahaemolyticus* in both *in vitro* and *in vivo* conditions, which results in increased resistance of *L. vannamei* against *V. parahaemolyticus* AHPND strain. Moreover, the aggregating bioflocs are rich in microbially bioactive components (so called MAMPs) which potentially improve the immune response resulting in better growth performance and improved resistance against pathogenic microbial infections (Cardona et al., 2015; Anand et al., 2017; Lee et al., 2017). For instance, the expression of immune related genes, i.e., prophenoloxidase, serine protease, prophenoloxidase activating enzyme, complement system and lysozyme, were reported to be increased significantly in *L. vannamei* cultured in a biofloc system leading to an increased resistance against *Vibrio harveyi* (Kim et al., 2014; Liu et al., 2017). In another study, Xu and Pan (2013) found that shrimp grown in biofloc environment has higher total haemocyte count, phagocytic activity, and total antioxidant activity as compared to non-biofloc control shrimp (Xu and Pan, 2013). Hence, further studies on the mechanisms by which bioflocs interfere with both host nutrition and immunological response on the one hand and expression of virulence genes in *V. parahaemolyticus* on the other hand will help to better understand how biofloc system enhance survival of *L. vannamei* upon *V. parahaemolyticus* AHPND strain challenge.

In conclusion, the findings presented here provide new insight on the protective action of biofloc system against *V. parahaemolyticus* AHPND strain. Our results imply that the protective effect of biofloc system is at least partially caused by steering the phenotype switching, where AHPND-causing *V. parahaemolyticus* might switch to a non-virulent matrix-enclosed biofilm phenotype. Furthermore, the biofloc-induced phenotype switching seems to be responsible for regulating the expression of AHPND bacteria flagella-related motility genes and Pir toxins and AHPND plasmid genes in both *in vitro* and *in vivo* conditions. The ability of the biofloc system to boost the water quality, growth performance, and resistance of *L. vannamei* against *V. parahaemolyticus* AHPND strain makes it a potent aquaculture technology that will be valuable to prevent microbial infection including AHPND and increase the shrimp production with high-density and minimal or no water exchange culture.

## Data Availability Statement

The raw data supporting the conclusions of this article will be made available by the authors, without undue reservation, to any qualified researcher.

## Author Contributions

VK and PB conceived and designed the experiments. VK performed the experiments and drafted the figures and manuscript. VK, MW, and TL made the laboratory analysis, statistics, and interpreted data. VK, MW, and PB reviewed and edited the manuscript. All the authors approved the final manuscript.

## Conflict of Interest

The authors declare that the research was conducted in the absence of any commercial or financial relationships that could be construed as a potential conflict of interest.
