# Supplementary material for: Biofloc-Based Enhanced Survival of Litopenaeus vannamei Upon AHPND-Causing Vibrio parahaemolyticus Challenge Is Partially Mediated by Reduced Expression of Its Virulence Genes
Source: Front Microbiol. 2020 Jun 24;11:1270. doi: 10.3389/fmicb.2020.01270 (PMC7326785; doi:10.3389/fmicb.2020.01270)
Supplement: Supplementary file 1 [file Data_Sheet_1.docx]

**Supporting information**


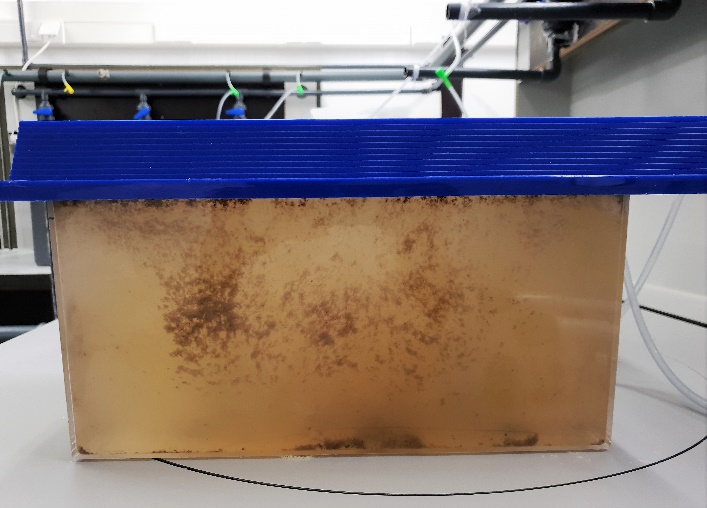

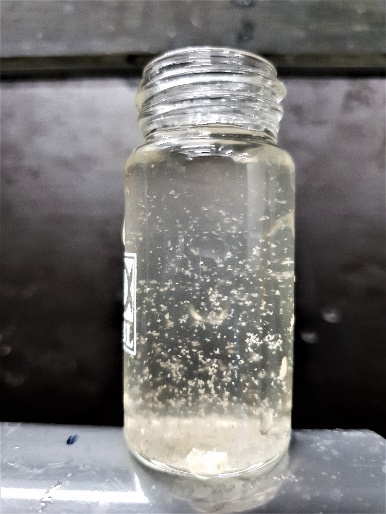


**Figure S1 Biofloc enriched suspension in seawater**

**Table S1 Specific primers and their sequence used for reverse transcriptase real-time PCR**

| Gene | Function |  | Primer sequence (5’ – 3’) | Product size (bp) | References |
| --- | --- | --- | --- | --- | --- |
| Flagella-related motility genes | | | | | |
| flaA | Polar flagellin | F | CTGCGGGTCTTCAAATCTC | 205 | Yang and Defoirdt 2015 |
|  |  | R | GTTAGTGGTCTCGTTCATTGC |  |  |
| fliS | Polar flagellin specific chaperone | F | CTCCGCACAAAGTCATTCAA | 224 | Yang and Defoirdt 2015 |
|  |  | R | CAATGTCACCACCATCTTCC |  |  |
| cheR | Chemotaxis protein | F | ATGCGATGACGACTAACGA | 174 | Yang and Defoirdt 2015 |
|  |  | R | ACGCTTGGCAATAAACCTG |  |  |
| Marker gene indicate presence of non-virulent AHPND *Vibrio parahaemolyticus* phenotype | | | | | |
| AlkPhoX | Alkaline phosphatase PhoX | F | GAACAAGATCGTATTGGTG | 254 | Kumar *et al.*, 2020 |
|  |  | R | CCAAACGCACAACCTTC |  |  |
| Virulent AHPND plasmid (responsible for toxin production) | | | | | |
| PirB^VP^ | Encodes PirB^VP^ toxin | F | ACTAGGCAAGGCTCATAAATATGACG | 102 | Han et al. 2015 |
|  |  | R | ATTGCTTCAGGTCCATTGGCAATAA |  |  |
| ORF-14 | Copy number of the pVA1 plasmid | F | GGCTCTTTCATAGGTGGTGTCATTC | 103 | Han et al. 2015 |
|  |  | R | CGACTACTATGCCGTTGAGTTGAAG |  |  |
| Reference gene | | | | | |
| toxR | Endogenous control | F | AATCCATGGATTCCACGCGTTATTT | 103 | Han et al. 2015 |
|  |  | R | CACCAATCTGACGGAACTGAGATTC |  |  |
| rpoA | RNA polymerase A submit (rpoA) (endogenous control) | F | CGTAGCTGAAGGCAAAGATGA | 197 | Yang and Defoirdt 2015 |
|  |  | R | AAGCTGGAACATAACCACGA |  |  |
